# Cyber Risk Management of API-Enabled Financial Crime in Open Banking Services

**DOI:** 10.3390/e28020163

**Published:** 2026-01-31

**Authors:** Odion Gift Ojehomon, Joanna Cichorska, Jerzy Michnik

**Affiliations:** 1 Department of Operations Research, Faculty of Informatics and Communication, University of Economics in Katowice, ul. 1 Maja 50, 40-287 Katowice, Poland; odion.ojehomon@edu.uekat.pl; 2 Department of Banking and Financial Markets, Faculty of Finance, University of Economics in Katowice, ul. 1 Maja 50, 40-287 Katowice, Poland; joanna.cichorska@uekat.pl

**Keywords:** open banking (OB), risk management, System Dynamics (SD), Agent-Based Modelling (ABM), Monte Carlo (MC) simulation, Application Programming Interface (API) security, Payment Services Directive (PSD2), third-party providers (TPPs), cyber risk, financial sector

## Abstract

Open banking reshapes the financial sector by enabling regulated third-party providers to access bank data through APIs, fostering innovation but amplifying operational and financial-crime risks due to increased ecosystem interdependence. To address these challenges, this study proposes an integrated risk-management framework combining System Dynamics, Agent-Based Modelling, and Monte Carlo simulation. This hybrid approach captures feedback effects, heterogeneous agent behaviour, and loss uncertainty within a simulated PSD2-style environment. Simulation experiments, particularly those modelling credential-stuffing waves, demonstrate that stricter onboarding thresholds, tighter API rate limits, and enhanced anomaly detection reduce operational tail losses by approximately 20–30% relative to baseline scenarios. Beyond these specific findings, the proposed framework exhibits significant universality; its modular design facilitates adaptation to broader contexts, including cross-border regulatory variations or emerging BigTech interactions. Ultimately, this multi-method approach translates complex open-banking dynamics into actionable risk metrics, providing a robust basis for targeted resource allocation and supervisory stress testing in evolving financial ecosystems.

## 1. Introduction

The foundations of modern open banking in Europe can be traced to the 2007 Payment Services Directive (PSD) [1], which sought to create a single market for payment services across the European Union. PSD established the legal basis for the Single Euro Payments Area (SEPA), strengthened consumer protection through improved transparency and liability rules, and promoted competition by allowing non-bank entities to provide regulated payment services. The revision of this framework through PSD2 in 2015 [2] marked a major regulatory shift: by enabling licensed third-party providers (TPPs) to access customer account data with explicit consent through standardised, secure Application Programming Interfaces (APIs), and mandating strong customer authentication, PSD2 catalysed the transition toward interoperable, API-driven financial services. This regulatory evolution facilitated a broader digital transformation of the banking sector. Traditional institution-centric architectures have increasingly given way to platform-based, data-driven ecosystems in which external service providers, fintechs, and technology firms play growing roles in customer interaction and financial intermediation [3,4]. Together, these developments laid the foundations for what is now widely referred to as Open Banking.

Open banking (OB) refers to the sharing and leveraging of customer-permissioned data by banks with third-party developers and firms to build applications and services, such as those that provide real-time payments, enhanced financial transparency for account holders, and new marketing and cross-selling opportunities [3]. It has become a central element of contemporary financial innovation. Across jurisdictions, OB is recognised as a catalyst for increased competition, financial transparency, and customer empowerment while simultaneously reshaping business models and market dynamics [5].

### Structural Changes Induced by Open Banking

Open banking has triggered several structural shifts in how financial services are organised, delivered, and governed. By mandating or enabling authorised third-party providers (TPPs) to access consumer-permissioned financial data through standardised APIs, open banking has reconfigured the competitive landscape, catalysed new business models, and accelerated digital transformation within the banking sector. This section synthesises the key trends and illustrates them with real-world examples that are directly enabled by open-banking frameworks.

Emergence of Data-Driven Banking Models: Open banking enables financial institutions and fintechs to leverage real-time, permissioned transaction data obtained via APIs to provide personalised financial services. Personal finance management (PFM) applications—such as Mint (US) and Yolt (EU)—use open-banking aggregation APIs to consolidate multi-bank information, generate spending insights, and deliver automated budgeting recommendations [6]. Similarly, European API aggregators like Tink provide banks with “Money Manager” modules that integrate categorisation algorithms and behavioural analytics directly into bank apps [7]. These examples demonstrate how API-enabled data access shifts value creation from traditional balance-sheet intermediation toward analytics-driven advisory models, reinforcing a broader transition toward data-centric banking.

Expansion of Fintech–Bank Collaboration and API Ecosystems: Mandatory API access has accelerated collaboration between incumbent banks and fintech firms. Banking-as-a-Service (BaaS) platforms—such as Solaris in Germany—provide regulated account, card, and payment modules via APIs, allowing non-bank brands to embed banking functionality without acquiring a banking licence [8,9]. These arrangements exemplify a shift from vertically integrated banking to modular ecosystem architectures, where banks specialise in regulated infrastructure while fintechs assume customer-facing roles. API-based interoperability thus becomes a strategic asset that determines partnership models, innovation speed, and cost efficiency.

API-Driven Embedded Finance and Real-Time Service Delivery: Open-banking Application Programming Interfaces (APIs) support embedded finance by enabling real-time access to account information, identity verification, and payment initiation. “Buy Now, Pay Later” (BNPL) providers such as Klarna use Payment Services Directive 2 (PSD2)-compliant APIs to perform instant affordability checks and verify bank-account ownership before issuing credit at checkout [10]. Platforms such as Shopify integrate banking functions: such as Shopify Balance, using financial APIs that rely on open-banking data flows and regulated Payment Initiation Services (PIS) [11]. In both cases, open-banking connectivity enables financial services to be embedded seamlessly into non-financial digital journeys, reducing friction and enhancing user experience.

Intensified Competition and Reduced Barriers to Market Entry: Open banking lowers traditional barriers to entry by granting TPPs access to customer account information that was previously exclusive to banks. This has enabled digital challengers, such as Monzo, Starling Bank, and Chase UK, to compete directly on user experience, data-driven features, and API-enabled service integration. These institutions consistently rank highly in Competition and Markets Authority (CMA) customer-satisfaction surveys, outperforming legacy banks on digital service metrics [12,13]. API-based account switching, multi-bank aggregation, and consent dashboards further reduce switching costs, intensifying competition across the sector.

Enhanced Consumer Empowerment and Data Portability: Open-banking legislation grants customers explicit rights to share financial data with authorised providers, enhancing data portability and enabling greater service customisation. Analyses by the Organisation for Economic Co-operation and Development (OECD) highlight how OB frameworks support “multi-homing,” improved price comparison, and reduced lock-in, as consumers combine services across multiple institutions [14,15]. Aggregators and comparison platforms use account-information Application Programming Interfaces (APIs) to generate personalised product recommendations, improving transparency and strengthening consumer bargaining power relative to traditional banks.

Transformation of Regulatory and Supervisory Approaches: Open banking has prompted regulators to update supervisory frameworks to account for data-sharing ecosystems and API intermediaries. In the European Union, PSD2 and the associated Regulatory Technical Standards (RTS) mandate strong customer authentication and secure communication protocols for APIs. The United Kingdom’s Open Banking Implementation Entity (OBIE) and Brazil’s phased open-finance framework combine regulatory mandates with industry-led standards to coordinate API specifications, liability rules, and data-governance requirements [16,17]. These shifts illustrate how supervisory priorities expand from prudential oversight to include cybersecurity, API reliability, and third-party risk management.

Shift Toward Embedded and “Invisible” Banking: As Application Programming Interface (API) infrastructures mature, financial services increasingly operate in the background of digital experiences. Ride-sharing applications such as Uber and e-commerce platforms integrate in-app wallets, instant payouts, and embedded credit products that rely on bank-to-platform API connections for account verification, identity checks, and settlement [18,19]. In Poland, the BLIK mobile payment system provides a related example: it is embedded directly into participating banks’ mobile applications and enables customers to make online and in-store payments, withdraw cash from automated teller machines (ATMs), and initiate person-to-person transfers using one-time codes generated within their banking app, rather than traditional card credentials [20,21]. Such solutions illustrate how Application Programming Interface (API)-enabled payment schemes—often building on open-banking infrastructures and strong customer authentication—allow financial services to become “invisible”—delivered contextually within broader digital ecosystems while still relying on regulated banking infrastructure.

Taken together, these trends demonstrate that open banking is not merely a regulatory requirement but a foundational driver of structural change in the financial sector. However, the transition from closed, institution-centric systems to highly interconnected, API-enabled ecosystems introduces new categories of risk. Increased reliance on APIs, distributed data-processing environments, and third-party dependencies heightens exposure to cybersecurity threats, privacy violations, operational disruptions, and governance failures [22]. Specifically, API-enabled financial crime manifests as fraud and operational loss events that arise from or are amplified by third-party access to bank accounts through open-banking APIs. These vulnerabilities affect multiple stakeholders. Customers face elevated risks of fraud, identity theft, and data misuse; banks confront greater operational complexity, compliance burdens, and reputational exposure arising from third-party failures; and the financial system as a whole may experience heightened concentration, cross-platform contagion, and systemic instability [23]. Regulatory fragmentation and legal tensions—most notably between PSD2’s data-access provisions and the General Data Protection Regulation (GDPR)—further complicate oversight, liability allocation, and cross-border harmonisation [24,25].

While existing research has examined individual dimensions of open-banking risk, such as cybersecurity, operational resilience, or third-party dependency [26,27], these aspects are typically analysed in isolation. Consequently, limited attention has been paid to how risks interact, propagate, and amplify within interconnected open-banking ecosystems. This fragmentation constitutes a significant research gap, as it remains unclear whether integrated and dynamic risk-assessment approaches can more effectively capture the multidimensional nature of open-banking risk.

Given the identified limitations, the central research question of this study is whether a unified methodological framework can systematically identify, quantify, and mitigate interdependent risks arising from technological, organisational, and regulatory sources in open banking. Accordingly, this article advances the following theses:

1. Open-banking regulation has accelerated the emergence of platform-based financial ecosystems characterised by increased data sharing and third-party participation.
2. These ecosystems generate novel and interdependent risk exposures for commercial banks that are not adequately captured by conventional risk-management approaches.
3. The multidimensional and dynamic nature of open-banking risks necessitates an integrated risk-assessment framework that explicitly accounts for feedback effects and cross-actor dependencies.
4. Multi-method modelling approaches are more effective than static, single-method techniques for evaluating risk propagation and mitigation in open-banking environments.

To address this problem, this study develops a hybrid risk-assessment framework integrating System Dynamics (SD), Agent-Based Modelling (ABM), and Monte Carlo (MC) simulation. SD is used to represent macro-level feedback mechanisms linking incident dynamics, security investments, user adoption, and ecosystem reliability. ABM captures heterogeneous interactions among banks, TPPs, customers, and adversarial actors, allowing for adaptive behaviour and micro-level risk propagation. MC simulation complements these components by modelling uncertainty and estimating loss distributions and tail-risk measures. Together, these methods provide an appropriate analytical basis for open-banking environments characterised by non-linear interactions, behavioural heterogeneity, and adversarial dynamics.

In its baseline form, the SD stocks and flows are explicitly *ecosystem aggregates*, which makes the model immediately useful for a scheme operator or regulator responsible for national open-banking policy (standards for controls, sector-wide throttling protocols, accreditation/onboarding rules, incident playbooks). The ABM and loss layers then translate those policies into operational KPIs (fraud-per-10k by TPP, incident backlogs, daily losses, VaR/ES), enabling stress testing of credential-stuffing waves and other shocks. With minor model variables indexing and simple mapping of losses to bank profit and loss (P&L), the same mechanics become a decision tool for institutional stakeholders such as the Chief Risk Officer (CRO), Head of Fraud Strategy, Chief Information Security Officer (CISO), or the Open Banking product owner. In that variant, the levers (rate limits, anomaly thresholds, onboarding criteria, control investment) are tuned against tail-risk targets and service constraints, while the ABM reveals which TPP connections dominate exposure. Thus, whether used by a national program owner or a specific bank, the model provides a transparent, calibratable bridge from policy choices to measurable risk outcomes under uncertainty.

Preliminary simulation results show that policy interventions—such as stricter TPP onboarding criteria, tighter rate limits, and higher anomaly-detection thresholds—reduce tail-loss exposure by approximately 20–30%, even when overall incident frequency declines only modestly. These findings indicate that integrated, multi-method modelling can effectively support risk-management decision-making in open-banking ecosystems. This study contributes to the literature by presenting a unified simulation-based framework that explicitly captures dynamic, cross-actor risk propagation and enables the evaluation of targeted resilience strategies.

The remainder of this paper is organised as follows. Section 2 reviews the literature on open-banking adoption and digitalisation. Section 3 examines the principal categories of risk inherent in open-banking ecosystems. Section 4 presents the integrated SD–ABM–MC framework. Section 5 details the simulation methods and parameter selection rationale. Section 6 describes the model verification and validation procedures. Section 7 presents the simulation design, specifically the ’credential-stuffing’ scenarios, and analyses the empirical results. Section 8 discusses the implications for policy and practice and also indicates directions for future research. Finally, key insights and conclusions are included in Section 9.

## 2. Literature Review

The academic literature addressing open banking and its associated risks spans multiple domains, including banking and finance, information systems, financial regulation, and risk modelling. Rather than conceptualising open banking solely as a technological development or a regulatory intervention, an expanding body of research frames it as a platform-based financial ecosystem characterised by extensive data sharing, third-party participation, and complex interdependencies among heterogeneous actors. This section synthesises the literature across four interrelated streams and identifies the research gap addressed by the present study.

### 2.1. Open Banking as a Platform-Based Financial Ecosystem

A first group of studies examines how access-to-account regulation and data sharing reshape the structure of financial intermediation. Vives [28] argues that digitalisation and mandated data access weaken banks’ informational advantages and shift competition toward data, distribution, and platform control. Boot et al. [29] similarly show that fintech-driven unbundling reduces switching costs and enables new entrants to compete in customer-facing segments, while incumbent banks increasingly specialise in regulated balance-sheet and infrastructure services. Thakor [30] surveys the fintech and banking literature and concludes that platform-based intermediation alters value creation and rent allocation across the financial-services value chain.

A second group of studies explains how this structural shift is enabled by modular technological architectures. Tiwana et al. [31] demonstrate that platform evolution depends on the joint design of architecture and governance, implying that interface standardisation is central to ecosystem growth. Eaton et al. [32] show how boundary resources such as APIs can be dynamically adjusted to balance third-party innovation with platform control. Karhu et al. [33] further document how platform owners use boundary resources strategically to manage openness and competitive threats in digital ecosystems.

Taken together, these studies support viewing open banking as an ecosystem-level transformation characterised by modular architectures, third-party participation, and interdependence among heterogeneous actors. This perspective departs from traditional bank–customer models and motivates analysing governance and risk at the ecosystem rather than the institutional level.

### 2.2. Regulatory and Governance Challenges in Open Banking

A stream of research focuses on how regulatory design structures participation and accountability in open-banking ecosystems. Zetzsche et al. [22] analyse data-driven finance regimes and argue that frameworks such as PSD2 pursue multiple objectives—competition, innovation, and consumer protection—while delegating substantial discretion to national authorities, resulting in heterogeneous implementation outcomes. Zetzsche et al. [34] further highlight governance challenges arising from the interaction between financial regulation and platform-based business models, particularly with respect to liability allocation among banks and third-party providers.

A related set of studies examines the legal interface between open-banking regulation and data protection law. Ferretti [35] characterises the relationship between PSD2 and the GDPR as an uncomfortable cohabitation, noting that overlapping requirements regarding consent and data use create uncertainty over responsibility for compliance and incident response. Gounari et al. [24] analyse PSD2 compliance in conjunction with cybersecurity standards and show that fragmented regulatory requirements complicate risk governance across organisational boundaries. Colangelo [36] draws comparative lessons from the EU experience and argues that regulatory-driven data sharing inevitably involves trade-offs between openness, consumer protection, and accountability.

Overall, these studies indicate that regulatory and governance arrangements play a central role in shaping not only access and competition in open banking but also the allocation of responsibility and ownership of risk across interconnected ecosystem participants.

### 2.3. Risk Exposure in API-Enabled Financial Systems

A number of studies examine how API-enabled intermediation and third-party integration affect operational and cyber risk in financial systems. Aldasoro et al. [37] show that the shift toward digital delivery and greater technological interconnectedness heightens exposure to cyber and technology-related operational risks. Barroso and Laborda [26] highlight that digitalisation in finance is associated with heightened cybersecurity, privacy, and operational risk concerns. Evidence from open-banking implementations indicates that dedicated-interface downtime and API availability issues can disrupt third-party access, and industry initiatives focus on improving API availability and performance across banks [38]. More broadly, reliance on critical third-party providers raises the possibility that a single disruption could affect multiple firms simultaneously through shared dependencies.

A related set of studies emphasises that such risks are not confined to individual institutions but may exhibit systemic properties. Aldasoro et al. [39] identify exposure concentration, shared infrastructure, and interdependencies as key drivers of cyber risk losses, consistent with the potential for cascading effects across interconnected actors. Danielsson et al. [40] argue that in highly interconnected financial systems, shocks are amplified through feedback mechanisms and non-linear dynamics, rendering traditional institution-centric risk assessments incomplete. These insights are particularly relevant for open-banking ecosystems, where shared APIs, identity providers, and data aggregators create common points of failure.

Taken together, these studies suggest that risk in open-banking environments is inherently interaction-driven: vulnerabilities arise not only from internal weaknesses but also from dependencies among banks, third-party providers, and shared digital infrastructure. This challenges risk-management frameworks that treat third-party failures as exogenous or peripheral.

### 2.4. Methodological Approaches to Interdependent Risk Modelling

A fourth group of studies addresses methodological approaches for analysing risk in systems characterised by interdependence, feedback, and uncertainty. Farmer et al. [41] critique traditional institution-centric stress testing frameworks, arguing that static scenario analysis fails to capture endogenous amplification mechanisms arising from interactions and behavioural responses in interconnected financial systems.

Agent-Based Modelling (ABM) has been widely applied to study financial systems with interacting actors. Farmer and Foley [42] argue that ABM is particularly well suited for analysing economies where aggregate outcomes emerge from micro-level interactions. Poledna and Thurner [43] demonstrate how network-based measures and agent-based simulations can be used to study systemic risk and cascading failure dynamics in financial networks. These tools are conceptually relevant for open-banking ecosystems, where heterogeneous actors interact through shared digital infrastructure.

In parallel, policy-oriented and supervisory studies highlight the importance of modelling tail-risk behaviour and uncertainty in digital financial infrastructures. European Central Bank [44] emphasises that cyber risk can pose threats to financial stability and that loss distributions are highly skewed and difficult to estimate using historical averages. Bank for International Settlements [45] underscore the need for forward-looking, scenario-based approaches to assess risks arising from fintech adoption and platform dependency.

Overall, while ABM, network models, and stochastic simulation techniques are well established individually, existing studies rarely integrate micro-level agent interactions, macro-level feedback structures, and tail-risk uncertainty within a unified modelling framework tailored to open-banking ecosystems.

### 2.5. Research Gap

Taken together, prior research provides important insights into the transformation of banking toward platform-based and API-enabled ecosystems, the regulatory and governance arrangements that structure participation in open banking, and the emergence of new operational and cyber risk exposures associated with increased interdependence. At the same time, the literature remains fragmented along both disciplinary and methodological lines.

Studies examining ecosystem restructuring and platform governance typically abstract from risk dynamics and propagation mechanisms. Conversely, research on operational and cyber risk often treats open-banking infrastructures and third-party dependencies in a stylised or institution-centric manner, without explicitly modelling the interactions among banks, third-party providers, and shared digital infrastructure. Similarly, methodological contributions applying agent-based models, network simulations, or stochastic techniques are rarely tailored to the specific architectural and regulatory features of open-banking environments.

As a result, there is limited empirical or analytical work that jointly captures (i) ecosystem-level feedback mechanisms induced by regulatory-driven openness, (ii) heterogeneous interactions among banks, third-party providers, and external service actors, and (iii) uncertainty and tail-risk behaviour arising from cyber and operational disruptions in API-enabled financial systems.

This study addresses this gap by developing an integrated System Dynamics–Agent-Based Modelling–Monte Carlo (SD–ABM–MC) framework to analyse risk propagation and mitigation in open-banking ecosystems.

## 3. Open-Banking Risks, Measures and Mitigations

### 3.1. Categorisation of Open-Banking Risks

The risks of open banking can be identified by the six following aspects: technical, financial crime and data, social, economic, regulatory and legal, and ethical and governance.

1. Technical Risks: These are risks linked to the design, security, interoperability, and resilience of open banking technology, particularly APIs, authentication systems, and integration with legacy infrastructure, such as variance in API standards across jurisdictions [46]; insecure fallback mechanisms such as screen scraping [36,47]; poor reliability of bank APIs, with high error rates and downtime [46]; clunky and inconsistent strong customer authentication [48]; and dependency risks from API aggregators [49].
2. Financial Crime and Data Risks: These are risks related to fraud, identity theft, data misuse, and other financial crimes that arise from increased data flows and third-party access enabled by open banking. They capture both technological vulnerabilities (such as insecure access and weak authentication) and behavioural risks (such as misuse of customer data by unauthorised or malicious actors). Examples include insecure fallback mechanisms such as screen scraping, which increases risks of impersonation, fraud, and data breaches [36,47]; consumer data being misused beyond the intended scope of consent [35]; systemic exposure to fraud as more third parties gain access to sensitive financial data [50]; and risks in credit markets where shared borrower data may enable exploitative practices, leaving consumers worse off [51]. In this paper, this category is operationalised through simulated credential-compromise shocks, fraud event generation at third-party providers, and the resulting distribution of financial losses.
3. Social Risks: These are risks arising from consumer behaviour, trust, adoption, and inclusion, reflecting how individuals interact with open-banking ecosystems, such as low consumer engagement and high loyalty penalties due to inertia [52]; confusion and frustration from complex consent and authentication flows [35]; reputational damage to incumbents from third-party failures [53]; and digital literacy and inclusion gaps, particularly for vulnerable or rural populations [50,52].
4. Economic Risks: These are risks tied to the financial and competitive consequences of open banking, including disintermediation, market structure shifts, and systemic volatility, such as disintermediation of banks and loss of bundling advantages [53]; excessive market power of BigTech and API aggregators [36,49]; high compliance and transformation costs with uncertain returns [54]; systemic risks from synchronised consumer behaviour through automated services [50]; and perverse outcomes in credit markets where all borrowers are worse off while industry profits rise [51].
5. Regulatory and Legal Risks: These are risks emerging from contradictions, ambiguities, and fragmentation in regulatory and legal frameworks governing open banking, notably PSD2 and GDPR, such as conflicting definitions of consent and portability in PSD2–GDPR interaction [35]; liability asymmetries where banks bear responsibility for TPP failures [47]; fragmented API standards and uneven enforcement across jurisdictions undermining interoperability [24,46]; and regulatory arbitrage between heavily regulated banks and lightly regulated fintechs [50].
6. Ethical and Governance Risks: These are risks concerning fairness, accountability, and transparency in open-banking ecosystems, including how data and market power are governed, such as ethical concerns around treating consumers as “data vectors” [54]; profiling and algorithmic discrimination in credit and insurance [50,55]; erosion of meaningful consent where consumers cannot understand or resist data use [35]; and governance tensions where platform operators or aggregators dictate access and competition [46].

While the six categories above provide a comprehensive overview of the open-banking risk landscape, this study focuses empirically on a specific subset of these risks. In particular, the modelling and simulation framework examines *financial crime and technical risks* arising from API-enabled third-party access, with an emphasis on credential-compromise and credential-stuffing attacks, the resulting fraud and operational incidents at third-party providers, and the associated aggregate financial losses. These risks are analysed through their interaction with key technical and governance controls, including API rate limits, anomaly-detection thresholds, and third-party onboarding requirements. Other risk categories—such as broader economic, social, regulatory, and ethical risks—are included in the taxonomy for contextual completeness but are not explicitly modelled as stochastic processes in the empirical analysis.

### 3.2. Risk Measures in Open Banking

In the growing literature on open banking, a wide range of risk measures have been proposed, spanning technical, operational, consumer, market, and systemic domains. This research emphasises those that are both consistently cited across studies and directly measurable in practice. These include indicators of API reliability and compliance (such as uptime, error rates, standards conformance), security and fraud prevention (such as Strong Customer Authentication pass rate, fraud rate, unauthorised transaction loss rate, incident reporting), consumer trust and engagement (such as transparency index, perceived risk score, adoption rate), and systemic stability (e.g., Herfindahl–Hirschman Index, bank Z-score, composite digital-risk indicator). Together, these metrics provide a cohesive framework for assessing the technical robustness, regulatory compliance, consumer protection, and financial stability of open-banking ecosystems.

Although the measures listed below span technical, consumer, and systemic dimensions of open banking, they are not intended to define new categories of bank risk. Rather, they operationalise established banking risk types within the specific context of API-enabled financial services. Consistent with standard banking practice, the measures can be grouped into operational risk, liquidity risk, credit risk, and solvency (bankruptcy) risk. Interest-rate risk is not modelled explicitly, as the framework abstracts from balance-sheet repricing effects and focuses on non-financial risks amplified by open- banking infrastructures. This classification allows the proposed indicators to be interpreted and used within existing bank risk-management structures.

Each risk measure is described below with its definition, mathematical formulation (where applicable), and references to the relevant literature.

#### 3.2.1. Operational Risk Measures

Operational risk is the primary focus of the framework, in line with the Basel definition of losses resulting from inadequate or failed internal processes, people and systems, or from external events [56]. In open banking, API dependencies and third-party access significantly amplify these risks.

1. API uptime: API uptime measures the proportion of time that banking APIs are operational and accessible. Low uptime undermines reliability and discourages adoption [46].
  $$\mathrm{Uptime}=100\times \left(1-\frac{\mathrm{Downtime}}{\mathrm{Total}\;\mathrm{time}}\right)$$
2. API Error Rate: the error rate indicates the proportion of failed API responses relative to total calls [46].
  $$\mathrm{Error}\;\mathrm{rate}=100\times \frac{\mathrm{Number}\;\mathrm{of}\;\mathrm{error}\;\mathrm{responses}}{\mathrm{Total}\;\mathrm{API}\;\mathrm{calls}}$$
3. Strong Customer Authentication (SCA) Pass Rate: SCA pass rate reflects the share of transactions successfully completed after Strong Customer Authentication [24,47].
  $$\mathrm{SCA}\;\mathrm{pass}\;\mathrm{rate}=100\times \frac{\mathrm{Successful}\;\mathrm{SCA}\;\mathrm{attempts}}{\mathrm{Total}\;\mathrm{SCA}\;\mathrm{attempts}}$$
4. Security and Operational Incident Rate: measures the frequency of reportable ICT or operational incidents related to open banking services [24].
  $$\mathrm{Incident}\;\mathrm{rate}=\frac{\mathrm{Number}\;\mathrm{of}\;\mathrm{reportable}\;\mathrm{incidents}}{\mathrm{Time}\;\mathrm{period}}$$
5. Fraud Rate: share of confirmed fraudulent transactions in relation to all transactions [47,48,50].
  $$\mathrm{Fraud}\;\mathrm{rate}\;\left(\mathrm{bps}\right)=10,000\times \frac{\mathrm{Fraudulent}\;\mathrm{transactions}}{\mathrm{Total}\;\mathrm{transactions}}$$
6. Unauthorised Transaction Loss Rate: captures the value of financial losses due to unauthorised transactions as a proportion of total transaction value [47].
  $$\mathrm{Loss}\;\mathrm{rate}=\frac{\mathrm{Value}\;\mathrm{of}\;\mathrm{unauthorised}\;\mathrm{transactions}}{\mathrm{Total}\;\mathrm{transaction}\;\mathrm{value}}$$
7. RTS/SCA/CSC Compliance Rate: proportion of PSD2 Regulatory Technical Standard (RTS) controls passed during testing [24,47].
  $$\mathrm{Compliance}\;\mathrm{rate}=100\times \frac{\mathrm{RTS}\;\mathrm{controls}\;\mathrm{passed}}{\mathrm{RTS}\;\mathrm{controls}\;\mathrm{tested}}$$
8. Standards Conformance: measures the extent to which APIs comply with published technical standards (e.g., Berlin Group) [24,47].
  $$\mathrm{Standards}\;\mathrm{conformance}=100\times \frac{\mathrm{Controls}\;\mathrm{satisfied}}{\mathrm{Controls}\;\mathrm{required}}$$
9. Composite Digital-Risk Indicator (CDRI): a composite index aggregating different technical, operational, and compliance risks into one score [57].
  $$\mathrm{CDRI}=\sum_{i=1}^{n}w_{i}z_{i}$$
  where $w_{i}$ are weights and $z_{i}$ are standardised risk indicators.

#### 3.2.2. Liquidity Risk Measures

Liquidity risk in the framework arises indirectly through the cash-flow impact of operational losses. Accordingly, liquidity-related exposure is captured by the unauthorised transaction loss rate (see Item 6) and the fraud rate (see Item 5), which quantify the magnitude and intensity of loss events that may trigger short-term funding stress.

#### 3.2.3. Credit Risk Measures

Open-banking-related incidents can indirectly affect credit risk by impairing borrower repayment capacity or generating exposures to third-party providers. The following measures capture these channels at an aggregate level.

Open-Banking Adoption Rate: share of eligible customers actively using open banking services [48,52,58].

$$\mathrm{Adoption}\;\mathrm{rate}=100\times \frac{\mathrm{Active}\;\mathrm{OB}\;\mathrm{users}}{\mathrm{Eligible}\;\mathrm{customers}}$$

Market Concentration (HHI): market concentration index measuring systemic risks of dominance by incumbents or BigTech [36,46].

$$HHI=\sum_{i=1}^{N}s_{i}^{2}$$

where $s_{i}$ is the market share of firm *i*.

#### 3.2.4. Solvency (Bankruptcy) Risk Measures

Solvency risk reflects the potential for accumulated losses to erode capital buffers and threaten bank stability. The framework captures this dimension using established stability indicators.

Bank Stability (Z-Score): a measure of bank solvency and risk of default [59].

$$Z=\frac{\mathrm{ROA}+\frac{\mathrm{Equity}}{\mathrm{Assets}}}{\sigma (\mathrm{ROA})}$$

Interest-rate risk is excluded from the analysis because the framework does not model asset–liability repricing or yield-curve dynamics, focusing instead on non-interest-related risks introduced by open-banking architectures.

### 3.3. Mitigation Strategies to Curb Open-Banking Risks

This subsection outlines some mitigation strategies that financial institutions, third-party providers, and regulators can employ to manage and reduce the risks inherent in open-banking ecosystems.

1. Technical risk mitigation: Technical exposures in open banking are primarily addressed through the deployment of secure, standardised APIs, the mandated move away from insecure practices such as screen scraping, and the adoption of Strong Customer Authentication (SCA) together with common-and-secure communication protocols [36,47]. These controls are embedded in PSD2/RTS interpretations and accompanying cybersecurity frameworks, as well as national implementation playbooks [24,60]. To reduce fragmentation and uplift baseline resilience, scholars and policymakers further recommend harmonised API specifications and oversight (such as performance/availability parity for TPP interfaces), coupled with certification and conformance regimes [46,61]. Banks are urged to ensure reliable integration between legacy cores and OB layers, invest in rigorous partner testing and platform curation, and adopt continuous monitoring (transaction/device analytics) and recognised standards such as ISO/IEC 27001 and PCI DSS [49,62,63]. Conceptual mappings of the field reinforce that regulation, platformisation, and data sharing jointly shape these technical safeguards [24,64,65].
2. Financial-crime and data-risk mitigation: Financial-crime and data-protection risks are mitigated through modernised, risk-based AML/KYC programs and the automation of screening, due diligence and ongoing monitoring with RegTech, alongside privacy-by-design safeguards (encryption, least privilege, access control) [64,66,67]. Clear licensing/accreditation and supervision of third-party providers, plus alignment with PSD2/GDPR and cybersecurity standards, reduce exposure to weakly governed actors and lower data misuse risk [24,36]. Evidence from PayTech development underscores the complementary role of supportive—but supervised—innovation tools such as regulatory sandboxes [48].
3. Social (consumer-protection) risk mitigation: Consumer risks are curbed by transparent, revocable consent; plain-language privacy statements; and user-centric consent journeys that reduce information asymmetries and build trust [36,58,67]. Foundational EU payment rules—liability limits for unauthorised use, single point of contact, and accessible dispute resolution—remain central to consumer protection as data sharing expands [68,69]. Sectoral studies highlight the importance of financial/digital literacy initiatives and trustworthy, permissioned data handling to enable safe adoption and sustained engagement [55,65,70].
4. Economic and market-structure risk mitigation: Market risks are mitigated by interoperable API standards that lower entry barriers, balanced monetisation models for sustainable API provisioning, and active platform governance that preserves quality while enabling innovation [16,46,49,61,63]. Formal analyses of credit competition under borrower data ownership show that voluntary sign-up equilibria, endogenous participation thresholds, borrower heterogeneity, and differentiated screening technologies can temper adverse selection and stabilise welfare [51]. Empirical and policy work further recommend incremental/experimental adoption, attention to technology spending discipline, and alignment of digital bets with banks’ diversification profiles to avoid fragility [55,59,71]. Cross-country evidence points to heterogeneous effects on traditional lending and PayTech growth, reinforcing the need for calibrated implementation [48,72].
5. Regulatory and compliance risk mitigation: Regulatory levers include harmonised legal frameworks that reconcile data protection, payments, and competition; clear liability allocation; mandatory licensing/supervision of TPPs [36,46,61,68,69]; and central coordination of standards and interoperability. PSD2/RTS provide the backbone for SCA, secure communications, interface obligations, and incident handling; complementary guidance ties these obligations to recognised cybersecurity frameworks and auditing practices [24,47,60]. National and EU-level policy also highlights sandboxes and supervisory dialogue to surface risks early without stifling innovation [48,55].
6. Ethics and governance risk mitigation: Ethical and governance concerns are managed through structured partner selection (such as, hybrid multi-criteria decision models), expert-weighted decision processes, and transparent platform rules for onboarding, certification, and quality control [49,62,63]. Privacy, identity, and accountability debates (such as SSI and data rights) motivate governance mechanisms that align incentives, deter discriminatory outcomes, and ensure effective enforcement for persistent non-compliance [55,66,73]. Collectively, these measures seek to balance innovation with rights, fairness, and societal trust across open-banking ecosystems [16,65].

### 3.4. Impacts of PSD2 and Similar Regulations on Banks and TPPs

This subsection analyses how the revised Payment Services Directive (PSD2) and comparable regulatory frameworks reshape the operational, strategic, and compliance environments of banks and third-party providers (TPPs).

1. Banks (Account Servicing Payment Service Provider (ASPSPs)): For banks (ASPSPs), PSD2 formalises “access-to-account” (XS2A) and requires dedicated or adapted secure interfaces plus Strong Customer Authentication (SCA), Common/Secure Communication, and incident reporting, which widens the security perimeter and compels investments in authentication, access control, monitoring, and resilience engineering [24,47] Standardisation choices shape operational risk: the UK’s prescriptive open-banking profiles and governance reduced interoperability frictions, whereas the EU’s more market-led approach produced uneven API quality that banks must mitigate via testing/certification, fallback interfaces, and robust third-party risk management [36,46,50]. Liability and reimbursement rules—together with the phasing-out of credential sharing/screen scraping—reallocate legal and reputational exposure and drive upgrades to fraud detection, dispute handling, auditability, and consent-lifecycle controls [47,68]. Because PSD2 sits alongside the GDPR, banks also face “legal knots” around lawful processing, minimisation, and consent scope, necessitating privacy-by-design, stronger due diligence over TPPs, and clearer disclosures [35,67]. Beyond compliance, mandated data mobility erodes incumbents’ information advantages and intensifies competition, so many banks pivot toward platform orchestration (“re-intermediation”) with tighter partner vetting and service-quality assurance, supported by RegTech and risk-based analytics to contain rising compliance costs [36,49]. Cross-border passporting and emerging open-finance proposals further expand oversight and interoperability challenges, reinforcing governance upgrades and operational risk controls [52,61].
2. Third-party providers (TPPs): For third-party providers (TPPs), PSD2’s licensing and supervision of account-information and payment-initiation services formalise market entry while imposing SCA/CSC, secure-interface use, and explicit, revocable consent aligned with GDPR principles of lawfulness, minimisation, and transparency [47,52]. Heterogeneous bank APIs and fragmented implementations translate into integration and reliability risks that TPPs address through interoperability tooling, certification, resilience practices, and clear compliance artefacts, even as policy discussions move toward common technical standards and sustainable API-monetisation models [36,46,50,61]. Access to consented transaction data can enhance screening and pricing but introduces “winner’s-curse” and adverse-selection frictions, prompting investments in rigorous data science, portfolio-risk controls, and trustworthy consent/user-experience design [51]. Privacy economics also implies that opt-in regimes may favour incumbents with established relationships, raising TPP acquisition costs and heightening the need for transparent notices and robust security to build trust [35,73]. Finally, supervisory tools such as regulatory sandboxes help TPPs test innovations under controlled conditions, while broader governance expectations, such as vendor management, auditability, and clear liability pathways, anchor operational resilience and consumer protection as the ecosystem scales [48,55].

While the preceding section outlines the principal risks associated with open banking, an effective response requires more than isolated control measures. In financial institutions, risk management constitutes a structured and continuous process of identifying, measuring, and mitigating risks in an integrated manner [74,75]. Such integration is particularly important in open-banking ecosystems, where technological, operational, and regulatory risks are interdependent and can propagate across multiple actors. Ensuring that these ecosystems remain secure, resilient, and compliant therefore necessitates a methodological framework capable of capturing these dynamic interactions. The following section develops this framework and details the approach used to operationalise risk-management principles within the analysis.

## 4. Conceptual Framework of the Hybrid Model

Complex financial systems exhibit interacting feedback loops, heterogeneous agent behaviour, and significant stochastic uncertainty, which are difficult to capture within a single modelling paradigm. Empirical and review studies have shown that System Dynamics (SD) and Agent-Based Modelling (ABM) address complementary levels of analysis—SD capturing aggregate feedback structures and ABM capturing micro-level decision processes—such that their joint application is often required when both macro-dynamics and heterogeneous agent behaviour shape system outcomes [76,77]. Recent work on multimethod simulation for risk management similarly argues that adding a stochastic component is necessary to represent uncertainty and tail-risk behaviour, and that SD–ABM–simulation hybrids provide richer and more policy-relevant insights than any single method alone [78].

**Figure 1:**
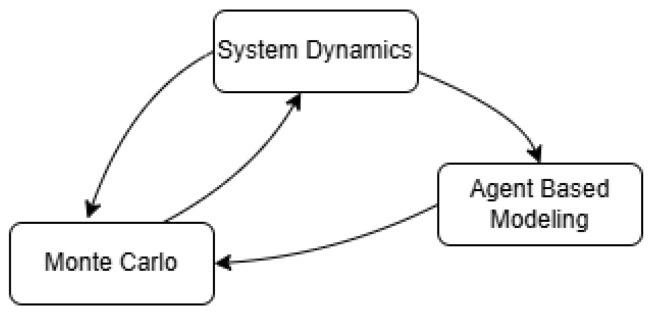
Model framework. Arrows represent the transfer of information between modules.

Building on these insights, the proposed framework integrates three complementary modelling approaches: System Dynamics (SD), Agent-Based Modelling (ABM), and Monte Carlo simulation, each contributing distinct analytical capabilities to capture feedback structures, heterogeneous agent interactions, and stochastic uncertainty within the system. In a first approximation of the comprehensive model, the connections between the component modules we propose are illustrated in Figure 1.

### 4.1. System Dynamics (SD)

In this model, the System Dynamics (SD) layer is purposefully framed at the ecosystem level: it aggregates behaviour across the open-banking program or market rather than representing any single institution. Stocks such as active users *U*, active TPPs *P*, control maturity *C*, incident backlog *I*, trust *R*, and budget *B* therefore capture sector-wide states, while flows encode how these states co-evolve under shared posture, demand, and threat conditions. This vantage point is chosen to reveal macro feedbacks—such as security–usability trade-offs, incident-driven trust erosion, and investment-led capability gains—that shape adoption, exposure, and resilience across the entire ecosystem. Bank-specific analyses can be obtained by switching to a bank-centric variant (indexing the same stocks by bank and treating the rest of the market as exogenous), but the baseline SD equations and feedback structure remain unchanged.

1. Users (number of active users)
  (1) $$\frac{dU}{dt}=\underset{\mathrm{User}\;\mathrm{adoption}\;\left(\mathrm{inflow}\right)}{\underset{︸}{\alpha \;R\;\max(U_{\max}-U,0)}}-\underset{\mathrm{User}\;\mathrm{attrition}\;\left(\mathrm{outflow}\right)}{\underset{︸}{\varphi \;\mathrm{Friction}(C)\;U}},$$
  where *t*—time, *R*—Reliability (variable), C∈[0,1]—Control Maturity Index (variable). It was assumed that the rate of increase in the number of users depends on the level of system reliability, while the friction resulting from excessive levels of control affects the rate of decline in the number of users.
  Auxiliary:
  $\mathrm{Friction}\left(C\right)=\frac{1}{2}\max{\left(0,C-0.5\right)}^{2}$—convex penalty function from stronger controls. The function Friction is assumed to be zero when the control maturity index *C* is below 0.5 and becomes active above.
  Parameters:
  α>0—user adoption rate; $U_{\max}$—the addressable market (ceiling) for open-banking active users; φ>0—sensitivity of users to friction.
2. Reliability (level of trust)
  (2) $$\frac{dR}{dt}=\underset{\mathrm{Service-driven}\;\mathrm{trust}\;\mathrm{gain}}{\underset{︸}{\psi \;\mathrm{Service}(C)}}-\underset{\mathrm{Incident-driven}\;\mathrm{trust}\;\mathrm{loss}}{\underset{︸}{\chi \frac{\lambda (t)}{\max(U,1)}}}$$
  where R∈[0,1], *C*—Control Maturity Index (variable). Credibility increases with the level of service and decreases with the intensity of incidents.
  Auxiliaries:
  $\mathrm{Service}\left(C\right)=1-\frac{1}{2}\max\left(0,C-0.9\right)$—quality/latency impact of controls; slightly lowering the service level if the control is too strong (*C* exceeds the value of 0.9).
  λ(t)—incident intensity; in the above equation, it is divided by the number of active users to get a fair, size-neutral exposure; it is defined by the following formula
  (3) $$\lambda \left(t\right)=AQ\left(t\right){\left(1-C(t)\right)}^{\eta }S\left(t\right),$$
  where *A*—base attack/contact factor; Q(t)—API call volume; ${\left(1-C(t)\right)}^{\eta }$—lowers incidents due to control maturity *C* (η>1); S(t)—seasonality/weekday factor.
  Q(t)—traffic volume is given by the following formula:
  (4) $$Q\left(t\right)=q_{0}\;U\left(t\right)\;\left(1+\kappa \;P(t)\right)\;\mathrm{Service}\;\left(C(t)\right).$$
  Parameters:
  ψ>0—translation of service quality into trust growth/recovery;
  χ>0—sensitivity of trust to incident exposure;
  $q_{0}>0$—calls/user/day;
  κ>0—marginal traffic lift per unit of P(t) (variable).
3. Control Maturity
  It is the combined capability across prevention, detection, and response for risks specific to open banking (API abuse, consent misuse, PISP (Payment Initiation Service Provider) fraud, scraping, DoS, supply-chain exploits). The single factor keeps the causal loop diagram readable and allows us to run broad policy “what-ifs” quickly.
  (5) $$\frac{dC}{dt}=\underset{\mathrm{Capability}\;\mathrm{gains}\;\mathrm{from}\;\mathrm{spend}}{\underset{︸}{\tau G\left(\mathrm{Spend}(B)\right)}}-\underset{\mathrm{Drift}/\mathrm{decay}\;\mathrm{of}\;\mathrm{controls}}{\underset{︸}{\delta_{C}}}$$
  Control Maturity Index *C* increases due to expenses on protecting the open-banking system and at the same time is subject to a natural weakening process.
  Auxiliaries
  *B*—budget stock available to security/controls.
  Spend(B)=ιB—the portion of *B* actually deployed into controls per unit time (could be capped).
  G(z)—concave gain function (diminishing returns).
  Parameters:
  τ>0—conversion rate from spend to maturity uplift; $\delta_{C}>0$—constant drift; ι>0.
4. Incident Backlog
  (6) $$\frac{dI}{dt}=\underset{\mathrm{Incident}\;\mathrm{inflow}}{\underset{︸}{\lambda (t)}}-\underset{\mathrm{Resolution}\;\mathrm{outflow}}{\underset{︸}{\mu (C)I}}$$
  The number of incidents increases proportionally to the intensity λ(t), which is defined in Equation (3). The ability to resolve incidents depends on the level of control C(t).
  Auxiliaries:
  λ(t)—incident intensity;
  $\mu \left(C\right)=\mu_{0}+\mu_{C}C$—resolution rate (increase with C).
  Parameters:
  $\mu_{0},\mu_{C}>0$.
5. Budget
  (7) $$\frac{dB}{dt}=\underset{\mathrm{Budget}\;\mathrm{inflow}}{\underset{︸}{\Pi_{\mathrm{in}}}}-\underset{\mathrm{Budget}\;\mathrm{outflow}}{\underset{︸}{\mathrm{Spend}(B)}},$$
  where $\Pi_{\mathrm{in}}$—a fixed per-day amount; here, as a first approximation, we assume that the budget is fed by a constant value. Similarly, expenditure was limited to cover control activities. In the future, for a more developed model, the constant value may be replaced by a variable and expenses may also include other items.
6. Third-Party Providers (TPP)—a count of active TPPs
  (8) $$\frac{dP}{dt}=\underset{\mathrm{Onboarding}/\mathrm{inflow}}{\underset{︸}{\rho_{\mathrm{onb}}\left[1-\mathrm{Friction}\left(C(t)\right)\right]}}-\underset{\mathrm{Offboarding}/\mathrm{outflow}}{\underset{︸}{\rho_{\mathrm{off}}\max\left(0,I\left(t\right)-I^{*}\right)}}$$
  The increase in the number of active TPPs is mitigated by the ‘Friction’ level. In turn, the reduction in the number of active TPPs is triggered when the incident rate exceeds a pre-determined comfort level.
  Auxiliaries
  $1-\mathrm{Friction}\left(C\right)=\frac{1}{2}\max{\left(0,C-0.5\right)}^{2}$—lower onboarding due to stronger controls.
  Parameters:
  $\rho_{\mathrm{onb}}>0$—baseline onboarding rate (TPPs/day); $\rho_{\mathrm{off}}>0$—pausing partnerships rate when operations are strained; $I^{*}$—service/operational comfort threshold.

#### 4.1.1. Feedback Loops

The system exhibits multiple interacting feedback loops. Reinforcing (positive) loops amplify growth dynamics, while balancing (negative) loops limit expansion or mitigate risk. Below, each loop is outlined with its causal chain and an interpretation.

##### Reinforcing Loops

(R1) User and Reputation Reinforcement by Investment

$$U\overset{\left(+\right)}{\to }B\overset{\left(+\right)}{\to }C\overset{\left(+\right)}{\to }R\overset{\left(+\right)}{\to }U$$

Interpretation: An increase in users (*U*) boosts the budget (*B*), enabling investment in control maturity (*C*), which enhances reliability (*R*). Improved reliability attracts more users, creating a self-reinforcing growth loop.

(R2) TPP Reinforcement by Investment

$$B\overset{\left(+\right)}{\to }C\overset{\left(+\right)}{\to }P\overset{\left(+\right)}{\to }B$$

Interpretation: Higher budget allows for control investments that raise third-party provider onboarding (*P*). Increased TPP participation brings additional service value and revenue, growing the budget further and reinforcing the cycle.

(R3) Growth by Risk Control

$$B\overset{\left(+\right)}{\to }C\overset{\left(-\right)}{\to }I\overset{\left(+\right)}{\to }R\overset{\left(+\right)}{\to }U\overset{\left(+\right)}{\to }B$$

Interpretation: Greater budget leads to improved controls, reducing incidents (*I*) and raising reliability (*R*). Higher reliability increases user adoption (*U*), further enlarging the budget. This loop demonstrates how control maturity supports sustainable growth through risk reduction.

##### Balancing Loops

(B1) Incidents Balancing Loop

$$U\overset{\left(+\right)}{\to }I\overset{\left(-\right)}{\to }R\overset{\left(-\right)}{\to }U$$

Interpretation: An expanding user base increases incidents (*I*), which lowers reliability (*R*). Reduced reliability discourages further user growth, forming a balancing feedback that limits uncontrolled expansion.

(B2) Balance by Friction

$$B\overset{\left(+\right)}{\to }C\overset{\left(-\right)}{\to }U\overset{\left(+\right)}{\to }B$$

Interpretation: Although budget increases enable improvements in controls (*C*), this raises user friction. Higher friction reduces user numbers (*U*), limiting budget growth. This loop shows the trade-off between security measures and user experience.

### 4.2. Agent-Based Modelling (ABM)

Agent-Based Modelling (ABM) simulates the behaviour and interactions of autonomous agents to observe emergent phenomena at the system level. In the context of open banking, ABM captures the dynamics of banks and third-party providers (TPPs), simulating transaction exposure, fraud attempts, adaptive throttling, and feedback updates. This micro-level model complements the System Dynamics (SD) layer and Monte Carlo (MC) simulation to produce a hybrid approach suitable for analysing risk propagation and control effectiveness in complex financial ecosystems [79].

#### 4.2.1. Time and Agents

The ABM runs in discrete time steps indexed by t=1,2,…,T, with each step representing one operational day (Δt=1 day). The agents are:

- *B* banks indexed by $b=1,\ldots ,B_{n}$;
- *P* third-party providers (TPPs) indexed by p=1,…,P;
- attackers represented as a single aggregate process with intensity multiplier $\iota_{t}$ and leak indicator $\ell_{t}$.

#### 4.2.2. Network Structure

The connections between banks and TPPs are represented by a bipartite adjacency matrix $M=\left[m_{b,p}\right]\in {\left\{0,1\right\}}^{B_{n}\times P}$, where:

(9) $$m_{b,p}=\left\{\begin{array}{cc} 1, & \mathrm{if}\;\mathrm{bank}\;b\;\mathrm{provides}\;\mathrm{API}\;\mathrm{access}\;\mathrm{to}\;\mathrm{TPP}\;p,\\ 0, & \mathrm{otherwise}. \end{array}\right.$$

Each connection is initialised stochastically, ensuring each TPP has at least one bank neighbour:

(10) $$m_{b,p}∼\mathrm{Bernoulli}\left(Pr_{\mathrm{connect}}\right),\;\mathrm{with}\;\sum_{b=1}^{B_{n}}m_{b,p}\geq 1\;\forall p,$$

where $Pr_{\mathrm{connect}}$ is the probability of connection.

1. Transaction Load Allocation: for traffic allocation, each TPP has an exposure proportional to its degree, normalised by the total number of connections:
  (11) $$c_{p}\left(t\right)=Q\left(t\right)\;\frac{d_{p}}{\sum_{p}d_{p}}$$
  where $d_{p}=\sum_{b=1}^{B}m_{b,p}$ is the TPP’s degree (number of connected banks).
2. Attack Attempts: given attack intensity $\lambda_{p}^{\mathrm{att}}>0$, the number of fraud attempts faced by TPP *p* is modelled as,
  (12) $$A_{p}\left(t\right)∼\mathrm{Poisson}\left(\lambda_{p}^{\mathrm{att}}\;c_{p}\left(t\right)\right),$$
  where attack intensity is given by
  $$\lambda_{p}^{\mathrm{att}}=k\cdot \iota_{p}\cdot \left(1+m\;l_{p}\right),$$
  Here, *k*—calibrating factor that depends on the scale of daily calls and puts the mean attempts into a numerically sensible 0.1–10 range before shocks. $\iota_{p}\in R_{\geq 0}$—attacker intensity multiplier is a continuous knob to sweep overall attacker pressure (scenario shock), $l_{p}\in \left\{0,1\right\}$—leak indicator multiplies attempts when a credential leak/wave is “on”, m>0—a leak multiplier (e.g., m=2).
3. Attack Success Probability: the probability that a single attack will be successful is given by a linear dependence on the control maturity index C(t), which is determined in the SD module. We assume this probability is limited to the interval [0.01,0.16]. The single-digit realised incidents/day per TPP under baseline attempt rates are consistent with early-stage operational data and with the SD incident scale.
  (13) $$Pr_{p}\left(t\right)=0.15\left(1-C(t)+0.01\right).$$
4. Realised Fraud: the number of successful fraud events $F_{p}\left(t\right)$ is
  (14) $$F_{p}\left(t\right)∼\mathrm{Binomial}\left(\lambda_{p}^{\mathrm{att}}\;c_{p}\left(t\right),Pr_{p}\left(t\right)\right).$$
5. Fraud Rate (Per 10,000 Calls): to compare TPP performance regardless of load, we propose the formula
  (15) $$\phi_{p}\left(t\right)=10^{4}\frac{F_{p}\left(t\right)}{\max\{c_{p}\left(t\right),1\}},$$
  if $c_{p}\left(t\right)>0$, otherwise $\phi_{p}\left(t\right)=0$.
6. Throttling Decision: a TPP is throttled if its fraud rate exceeds a threshold $K_{\mathrm{thr}}$;
  (16) $${\mathrm{throttle}}_{p}\left(t\right)=\left\{\begin{array}{cc} 1, & \phi_{p}\left(t\right)\geq K_{\mathrm{thr}},\\ 0, & \mathrm{otherwise}, \end{array}\right.$$
  and
  (17) $$H\left(t\right)=\sum_{p=1}^{P}{\mathrm{throttle}}_{p}\left(t\right)$$
  counts the number of throttled TPPs.
7. Adaptive Reallocation: throttled TPPs lose a traffic share fraction ρ∈(0,1] on the next day;
  (18) $$w_{p}\left(t+1\right)=w_{p}\left(t\right)\left(1-\rho \;{\mathrm{throttle}}_{p}\left(t\right)\right),$$
  followed by normalisation:
  (19) $$w_{p}\left(t+1\right)\leftarrow \frac{w_{p}\left(t+1\right)}{\sum_{\ell =1}^{P}w_{\ell }\left(t+1\right)}.$$
8. Aggregated Fraud:
  (20) $$F_{\mathrm{tot}}\left(t\right)=\sum_{p=1}^{P}F_{p}\left(t\right),$$
  which feeds back into the SD layer and into a Monte Carlo loss model.
9. Parameter Feedback Update:
  Control maturity C(t) and reliability R(t) from the SD layer affect attack intensity and success rates;
  (21) $$Pr_{0}\left(t+1\right)=Pr_{0}\left(t\right)\;f\left(C\left(t\right)\right),\;\lambda_{\mathrm{att}}\left(t+1\right)=\lambda_{\mathrm{att}}\left(t\right)\;g\left(R\left(t\right)\right).$$
  Here, f(C) is decreasing in *C* (improved controls reduce success probability), while g(R) increases with *R* (higher reliability attracts more adversarial focus).

### 4.3. Monte Carlo Model

The Monte Carlo method represents the stochastic core of the integrated SD–ABM–MC framework, transforming discrete incident counts into a full loss distribution from which tail-risk measures are derived. Monte Carlo simulation is widely used in financial risk management to estimate Value-at-Risk (VaR) and Expected Shortfall (ES) when loss distributions are complex or analytically intractable [80]. In operational and cyber-risk contexts, it is commonly combined with frequency–severity models to quantify aggregate losses and support capital adequacy assessment [81].

#### 4.3.1. Severity Process

The conceptual framework of this module rests on a bifurcated modeling approach that distinguishes between typical and systemic operational modes. The typical mode characterises the baseline operational environment, defined by idiosyncratic, low-impact incidents such as routine fraud, credential testing, and minor processing errors. Statistically, these events follow a lognormal distribution with moderate dispersion and minimal cross-sectional dependence, allowing standard Security Operations Center (SOC) capacities to manage them effectively. Because the “blast radius” of these incidents is inherently limited, they contribute to the aggregate loss frequency but rarely influence the extreme tail metrics of the risk profile.

In contrast, the systemic mode accounts for episodic regimes triggered by shared drivers, such as API logic flaws, large-scale aggregator outages, or coordinated social engineering campaigns. Unlike the typical mode, this regime is defined by high correlation across multiple edges and the potential for positive feedback loops, often leading to operational saturation. By employing a mixture of lognormal distributions, the model effectively captures the “fat tails” of the loss distribution—specifically VaR (Value-at-Risk) and ES (Expected Shortfall)—without the risk of overfitting. This dual-mode architecture provides superior pedagogical and analytical clarity; it allows for the separation of control levers, distinguishing between routine hygiene measures and the strategic playbooks required to suppress large-scale regime shifts.

Each event’s loss severity is drawn from a heavy-tailed distribution:

(22) $$X_{i}\left(t\right)∼\mathrm{Lognormal}\;\left(\mu_{t},\;\sigma \right),$$

with $\mu_{t}^{\mathrm{typ}}$, $\sigma^{\mathrm{typ}}$ for typical mode and $\mu_{t}^{\mathrm{sys}}$, $\sigma^{\mathrm{sys}}$ for systemic mode.

#### 4.3.2. Frequency Process

The daily event count is taken from the day’s realised incidents (currently from ABM), i.e., $N\left(t\right)=F_{\mathrm{tot}}\left(t\right)$. Given N(t) events, we draw the number of systemic-mode events

(23) $$N^{\mathrm{sys}}\left(t\right)∼\mathrm{Binomial}\left(N\left(t\right),\;p_{\mathrm{sys}}\right),$$

where $p_{sys}$ is small probability of systemic events, and set $N^{\mathrm{typ}}\left(t\right)=N\left(t\right)-N^{\mathrm{sys}}\left(t\right)$.

#### 4.3.3. Compound Loss Process

We simulate losses by drawing two independent batches:

- $N^{\mathrm{typ}}\left(t\right)$ draws from $\mathrm{Lognormal}(\mu^{\mathrm{typ}},\sigma^{\mathrm{typ}})$;
- $N^{\mathrm{sys}}\left(t\right)$ draws from $\mathrm{Lognormal}(\mu^{\mathrm{sys}},\sigma^{\mathrm{sys}})$,

and sum them together. Mathematically, it means for each event *i*:

(24) $$X_{i}\left(t\right)∼\left\{\begin{array}{cc} \mathrm{Lognormal}(\mu^{\mathrm{sys}},\sigma^{\mathrm{sys}}), & \mathrm{with}\;\mathrm{probability}\;p_{\mathrm{sys}},\\ \mathrm{Lognormal}(\mu^{\mathrm{typ}},\sigma^{\mathrm{typ}}), & \mathrm{with}\;\mathrm{probability}\;1-p_{\mathrm{sys}}. \end{array}\right.$$

Finally, the total financial loss is modelled as a compound frequency–severity process:

(25) $$\ell \left(t\right)=\sum_{i=1}^{N(t)}X_{i}\left(t\right),$$

where ℓ(t) is the total loss on day *t* and $X_{i}\left(t\right)$ is the monetary severity of the *i*-th event given by Equation (24).

#### 4.3.4. Risk Measure Estimation

From the simulated daily loss distribution, tail-risk measures are computed.

Value-at-Risk (VaR).

For confidence level q∈(0,1):

(26) $${\mathrm{VaR}}_{q}\left(t\right)=\inf\left\{x:P\left(\ell \left(t\right)\leq x\right)\geq q\right\}.$$

Expected Shortfall (ES).

(27) $${\mathrm{ES}}_{q}\left(t\right)=E\left[\ell \left(t\right)|\ell \left(t\right)>{\mathrm{VaR}}_{q}\left(t\right)\right].$$

A full list of model variables and parameters is provided in the Appendix A.

## 5. Simulation Methods, Parameters, and Justification

Certain input parameters were assigned arbitrarily, albeit within a realistic range. This approach was necessitated by the lack of available data and the ease with which these values can be adjusted for specific practical use cases.

Time and integration.

For the purpose of conducting the model simulation, we implemented the algorithms using Python 3.11.4. The model runs in discrete daily time (Δt=1 day), using forward-Euler updates for SD stocks and one-step sampling for ABM and loss generation. Randomness is produced by NumPy’s default_rng with a fixed seed set in the runner.

### 5.1. SD Module

#### 5.1.1. Users, See Equation (1)

$U_{\max}=10^{6}$ was set arbitraly. The nitial value $U\left(0\right)=10^{5}$ was set to represent an ecosystem where roughly 10% of the addressable population is initially active—large enough to generate meaningful API volume but small relative to market potential. This aligns with early-stage program penetration while leaving headroom for adoption dynamics. α=0.006 with daily steps implies a baseline logistic-like pull toward $U_{\max}$ modulated by trust R(t). For R(t)≈0.8 (the default), the effective rate is ≈0.0048 per day. This ensures smooth trajectories and avoids unrealistically fast saturation. φ=0.001 multiplies a convex friction term that is zero until controls exceed 0.5 and then grows quadratically. The magnitude keeps net adoption positive under reasonable *C* while still allowing strong controls (e.g., C>0.8) to create visible drag if trust is low. This reflects the small but non-negligible usability cost from tightening measures (step-up authentication, additional checks).

#### 5.1.2. Reliability, See Equation (2)

Initial condition R(0)=0.8 represents a mature, generally trusted program at baseline, leaving headroom for deterioration during stress and recovery under calm conditions. χ=0.02 is set larger than ψ=0.01 so that negative shocks (incidents) erode trust faster than routine good service replenishes it—consistent with observed customer behaviour and operational risk experience.

#### 5.1.3. Incident Intensity, See Equation (3)

Base attack factor $A=10^{-7}$ puts λ(t) in the ballpark of sub-daily to few-per-week incidents before control attenuation and seasonality, aligning SD intensity with ABM-realised incidents and keeping downstream loss sampling in a realistic range. Weakly seasonality is given by the function $S\left(t\right)=1+0.1\sin\frac{2\pi t}{7}$. S(t) injects predictable cadence (staffing patterns, user activity, attacker timing) without dominating policy effects. ±10% keeps seasonal movement visible but not overwhelming. With η=1.3, early gains in maturity deliver larger risk reductions than late-stage refinements—i.e., diminishing returns as best practice is approached. This reflects operational reality: basic hygiene removes a lot of risk; squeezing the last 10–20% is progressively harder and less impactful per unit. The exponent is “mild” to avoid driving $\lambda_{t}$ unrealistically close to zero at moderate control index *C*.

#### 5.1.4. API Volume, See Equation (4)

$q_{0}=3.0$—baseline calls per active user per day. Making Q∝U preserves units (calls/day) and yields intuitive, stable growth as the user base expands. The factor (1+κT) with κ=0.10 encodes that a richer ecosystem stimulates usage (more consent flows, features). Service(C) imposes no penalty through typical maturity (up to C=0.9) and only a mild linear penalty beyond that. This reflects that very strict controls (e.g., repeated step-ups) can depress throughput slightly but should not dominate exposure dynamics. Initial value Q(0)=0.0.

#### 5.1.5. Control Maturity, See Equation (5)

Spend(B)=min(0.01B,5000)—at most 1% of budget or 5k/day (capped version). $G\left(z\right)=5\cdot 10^{-6}\max\left(z,0\right)$—the absolute scale is tuned so that plausible daily spend (hundreds to a few thousand) can move *C* over weeks—not instantly. τ=0.02 keeps daily increments modest and numerically stable. A small daily decay $\delta_{C}=0.002$ prevents unrealistically permanent peaks: maturity erodes without upkeep (rules drift, patches lag, staff churn). This creates a realistic need for sustained investment rather than one-off boosts. Initial value C(0)=0.6.

#### 5.1.6. Incident Backlog, See Equation (6)

Incident inflow is given by incident intensity λ(t), which was discussed above. A linear map ($\mu \left(C\right)=\mu_{0}+\mu_{C}C$) is easy to explain and fit. $\mu_{0}=0.03$ provides baseline clearance of 3% of the backlog per day even at low control maturity (triage, automated rules, regular monitoring). $\mu_{C}=0.2$—high level of control (close to 1) adds about 20% per day of clearance capacity. Initial value I(0)=10.0.

#### 5.1.7. Budget, See Equation (7)

The budget stock has a simple inflow–outflow structure: a steady inflow (typical of an approved run-rate) and a state-dependent spend that scales with available funds but is capped to avoid unrealistic daily burn. Budget inflow $\Pi_{\mathrm{in}}$ is set at a fixed value of 1000 (currency/day). Spend(B)=min(0.01B,5000)—pend rule: up to 1% of current budget but no more that 5000/day. Initial value: B(0)=200,000.

#### 5.1.8. TPP, See Equation (8)

TPP count moves through (i) a baseline onboarding flow that is modestly dampened by strong controls (onboarding friction) and (ii) an offboarding (or suspension) flow that rises only when operational strain is high (backlog beyond $I^{*}=20$). This aligns with real programs: stricter controls can slow accreditation and ramp-up, and operational turmoil (spikes in unresolved incidents) can lead to temporary suspensions or voluntary exits. $\rho_{\mathrm{onb}}=0.02$, $\rho_{\mathrm{off}}=0.005$, P(0)=25.

### 5.2. ABM Module

#### Agents and Network Structure

For an initial testing, we chose the number of banks $B_{n}=3$, the number of TPP P=25, and probability of connection $Pr_{\mathrm{connect}}=0.5$. The 3 × 25 configuration represents an optimal pragmatic balance: it provides sufficient structure to evaluate policies and observe heterogeneity while remaining computationally efficient for rapid iteration and transparent diagnostics.

1. Transaction Load Allocation: no additional parameters to set.
2. Attack Attempts: $k=10^{-6}$—coincides with daily calls that can be 10^5^–10^6^ per TPP, $\iota_{p}=1$—baseline scale, $l_{p}=1$—no surge by default, m=2—triples attempts when a credential leak/wave is “on”.
3. Attack Success Probability: no additional parameters to set.
4. Realised Fraud: no additional parameters to set.
5. Fraud Rate (Per 10,000 Calls): no additional parameters to set.
6. Throttling Decision: the threshold $K_{\mathrm{thr}}=3.0$ and the number of TPPs that exceed the threshold is calculated (can be reported as KPI).
7. Adaptive Reallocation: at the initial stage of simulation, no changes are made to the allocation.
8. Aggregated Fraud: no additional parameters to set.
9. Parameter Feedback Update: this option is currently unused; it may be implemented in a more advanced model.

### 5.3. Monte Carlo Module

The severity component of the proposed model is calibrated using a two-mode lognormal mixture to ensure that the stochastic properties of the loss distribution align with empirical operational realities. The typical mode, parameterised by $\mu^{\mathrm{typ}}=9.0$ and $\sigma^{\mathrm{typ}}=1.1$, represents the baseline frequency of routine operational incidents, such as chargebacks and minor processing errors. With a median loss of approximately $8.1\cdot 10^{3}$ and a mean of $1.5\cdot 10^{4}$ (coefficient of variation ≈1.53), this setting provides a realistic scale and dispersion for day-to-day “noise.” These parameters are selected for their numerical stability, allowing for straightforward recalibration as historical loss data accumulates, while maintaining a clear distinction from tail-event dynamics.

The systemic mode is defined by a higher scale and heavier dispersion, with $\mu^{\mathrm{sys}}=12.0$ and $\sigma^{\mathrm{sys}}=1.2$. This shift places the median loss at approximately $1.6\cdot 10^{5}$, effectively positioning systemic events one to two orders of magnitude above the routine baseline. To capture the infrequent but high-impact nature of these regimes—such as large-scale credential dumps or aggregator outages—a mixture weight of $p_{\mathrm{sys}}=0.02$ is applied. This 2% probability ensures that systemic shocks meaningfully influence tail-risk metrics, specifically Value-at-Risk (VaR) and Expected Shortfall (ES), without distorting the mean during periods of operational stability. Furthermore, this parameterisation offers high interpretability for stress testing; researchers can simulate “wave” scenarios or loss clustering by incrementally adjusting the systemic weight (e.g., to 4% or 8%) or the location parameter $\mu^{\mathrm{sys}}$, providing a robust framework for assessing institutional resilience.

## 6. Model Verification and Validation

### 6.1. Conceptual Model Validity

This was assessed following the framework proposed by Sargent [82], which defines validation as evaluating whether the assumptions, structure, and causal relationships of a model are reasonable for its intended purpose. Given the limited observability of real-world open-banking fraud and operational-risk processes, validation focused on the internal coherence and plausibility of the model structure rather than on empirical calibration.

**Figure 2:**
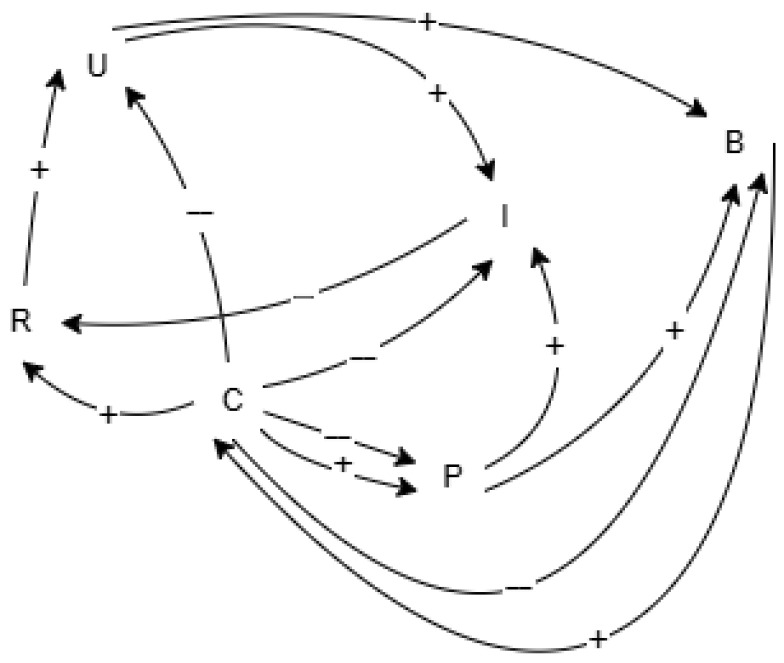
Causal loop diagram.

The conceptual structure of the hybrid SD–ABM–MC framework is illustrated in Figure 1, which presents the high-level integration of the System Dynamics, Agent-Based, and Monte Carlo components, and in Figure 2, which depicts the core causal feedback loops governing adoption, control maturity, incident dynamics, trust, and third-party participation. These diagrams were used as the primary artefacts for conceptual validation, enabling explicit inspection of the model’s causal logic and feedback structure.

The structural assumptions—such as the effectiveness of security controls in reducing attack success, the trade-off between control strength and user friction, adaptive attacker behaviour, and heavy-tailed loss severity—were identified and examined for plausibility relative to established theories and empirical findings in operational risk, cyber risk, and open-banking research. Directional causal relationships shown in the causal loop diagram (Figure 2) were explicitly inspected to ensure logical consistency; sign reversals were evaluated and rejected where they implied implausible system behaviour (such as higher control maturity increasing incident rates or incident growth improving trust).

Conceptual extreme-condition reasoning was applied to verify that the model structure enforces necessary limiting behaviour. In particular, the framework ensures that zero attack intensity implies zero incidents, zero adoption implies negligible API exposure, and near-maximal control maturity implies minimal attack success probability. Finally, consistency across the System Dynamics, Agent-Based, and Monte Carlo components was examined to confirm that shared concepts, such as incidents, controls, and exposure, have uniform interpretation and compatible time scales throughout the hybrid model.

Together, these checks support the conclusion that the conceptual model structure is internally consistent and sufficiently valid for its intended use in comparative risk-management and policy-analysis applications, in line with Sargent’s validation principles.

For the agent-based component, these conceptual checks correspond to agent-level and model-level validation within the Hierarchical ABM Validation framework proposed by He [83].

### 6.2. Computerised Model Verification

This was conducted to ensure that the implemented code correctly represents the conceptual model structure. Following [82], verification focused on confirming that the model logic, agent behaviours, and state-update mechanisms were implemented as intended. This included structured walkthroughs of the simulation logic, trace-based inspection of selected agent interactions, range and consistency checks on major variables, and the use of fixed random seeds to ensure reproducibility of stochastic components. These checks provide confidence that the computational implementation faithfully reflects the conceptual model design.

### 6.3. Operational Validity

This concerns whether the model’s output behaviour is sufficiently reasonable for its intended purpose. Following Sargent [82], operational validity was assessed through systematic exploration of model behaviour rather than through direct statistical comparison with real-system outputs, as comprehensive and observable data on open-banking fraud and operational losses are not publicly available.

Operational validation was conducted using a combination of extreme-condition simulation tests and sensitivity analysis. These experiments were designed to examine whether the implemented model responds plausibly and consistently to changes in key control and threat parameters, in accordance with its conceptual structure.

Extreme-condition tests were performed by examining model behaviour under limiting scenarios, such as near-zero control maturity, high control maturity, and varying levels of attacker intensity. The model exhibited qualitatively consistent behaviour across these scenarios, with weaker controls leading to increased incident rates and losses, and stronger controls resulting in reduced risk exposure, as expected from the underlying model logic.

In addition, sensitivity analysis was conducted by varying key policy and threat parameters across plausible ranges while holding other parameters constant. The resulting outputs demonstrated stable and intuitive responses, with no implausible or numerically unstable behaviour observed. These results indicate that the model’s behaviour is robust to parameter variation and suitable for comparative risk-management analysis.

**Figure 3:**
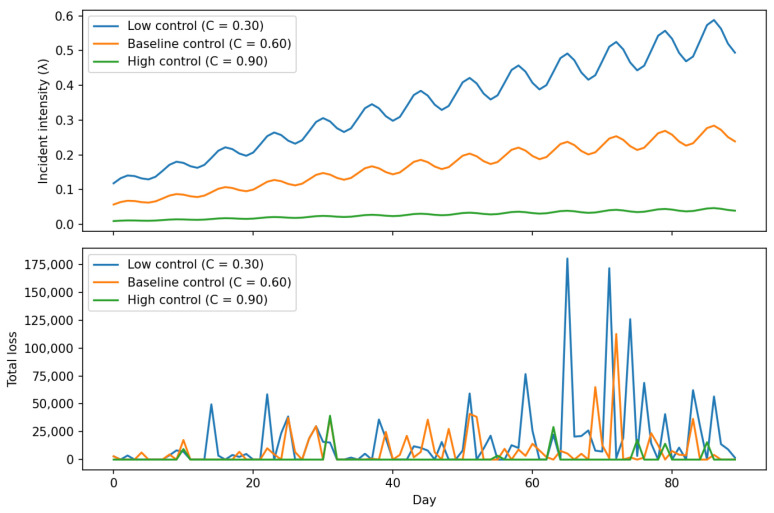
Extreme-condition operational validation of the model under low, baseline, and high control maturity regimes. Reduced control maturity leads to persistently higher incident intensities and heavier-tailed loss outcomes, while stronger controls suppress both incident generation and loss exposure, confirming that the model enforces the expected boundary behaviour implied by its conceptual structure.

**Figure 4:**
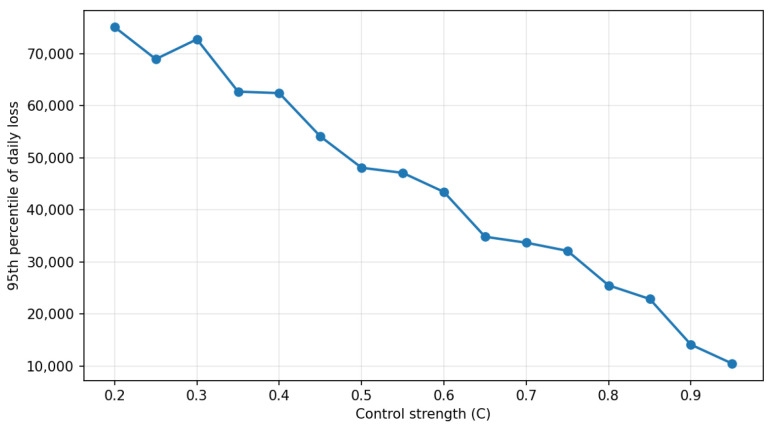
Sensitivity analysis of key control parameters. The figure shows the response of tail-risk metrics to gradual changes in control strength, demonstrating stable and monotonically decreasing risk reduction as controls are strengthened. The absence of discontinuities or counterintuitive behaviour supports the robustness of the model under parameter variation.

**Figure 5:**
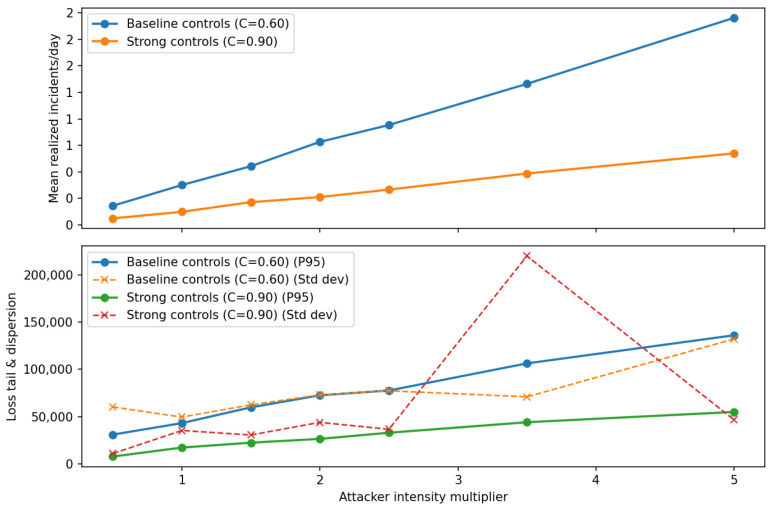
Threat-intensity response under fixed control configurations. Model response to increasing attacker intensity under fixed control configurations. Higher attacker intensity leads to increased incident rates and loss dispersion, while stronger controls dampen but do not eliminate risk exposure, confirming a plausible stress-response behaviour.

From an agent-based modeling perspective, these behavioural experiments correspond to output-level validation within the Hierarchical ABM Validation framework proposed by He [83]. The results of the operational validation experiments are presented in Figure 3, Figure 4 and Figure 5.

### 6.4. Data Validity and Confidence Level

This concerns the appropriateness, quality, and limitations of the data used to parameterise and evaluate the model. Following Sargent [82], it is explicitly acknowledged that comprehensive, high-quality empirical data on open-banking fraud incidents, third-party operational failures, and associated loss distributions are not publicly available due to confidentiality constraints, under-reporting, and regulatory disclosure limitations. As a result, several model parameters were specified using plausible ranges informed by the prior literature, regulatory guidance, and stylised characteristics of operational and cyber risk, rather than through direct empirical calibration.

Given these data limitations, a high level of confidence in pointwise predictive accuracy is neither claimed nor required for the intended purpose of the model. Instead, confidence in the model is derived from its structural coherence, behavioural plausibility, and robustness under systematic parameter variation, as demonstrated through the conceptual and operational validation steps. The model is therefore considered sufficiently valid for comparative risk analysis, scenario exploration, and policy-oriented evaluation of control strategies within open-banking ecosystems, while not intended for precise forecasting of institution-specific losses or regulatory capital requirements.

This explicit characterisation of data validity and confidence level ensures transparency regarding the scope of applicability of the proposed framework and is consistent with Sargent’s guidance that model credibility should be evaluated relative to both data availability and intended use.

## 7. Initial Model Testing

**Figure 6:**
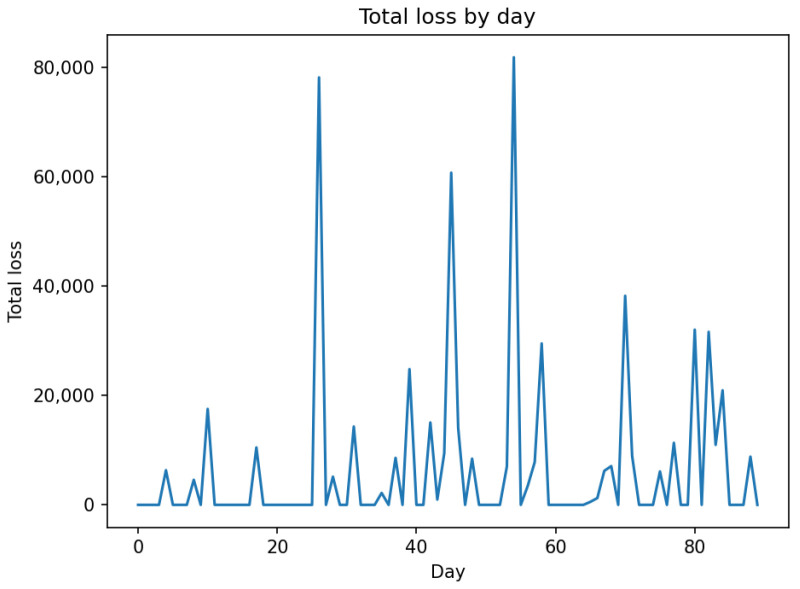
Total loss—default.

To demonstrate the model’s functionality, the “credential-stuffing wave” scenario was selected. This scenario represents a large-scale, automated cyberattack wherein adversaries leverage lists of compromised credentials to attempt unauthorised authentication across unrelated systems. By exploiting the prevalence of password reuse, these attacks frequently result in widespread Account Takeovers (ATOs). The model is initiated with the parameter values presented in Table 1. The horizon of simulations has been chosen to be 90 days. The values of policy levers and shock parameters for the default scenario, credential stuffing wave, and managerial reaction are presented in Table 2 and Table 3, respectively. Figure 6 shows the simulation of Total Loss in the default state. For simplicity, in the initial tests, throttling policy is not applied ($\phi_{p}\left(t\right)=0$).

**Figure 7:**
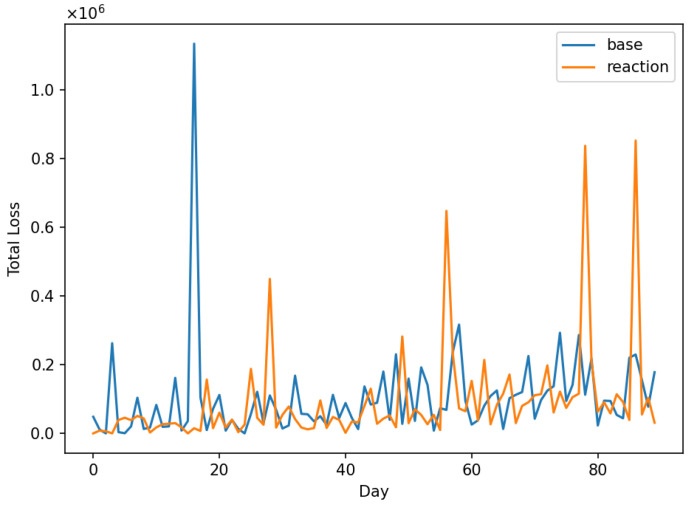
Total loss: comparison of the shock and reaction to the shock.

**Figure 8:**
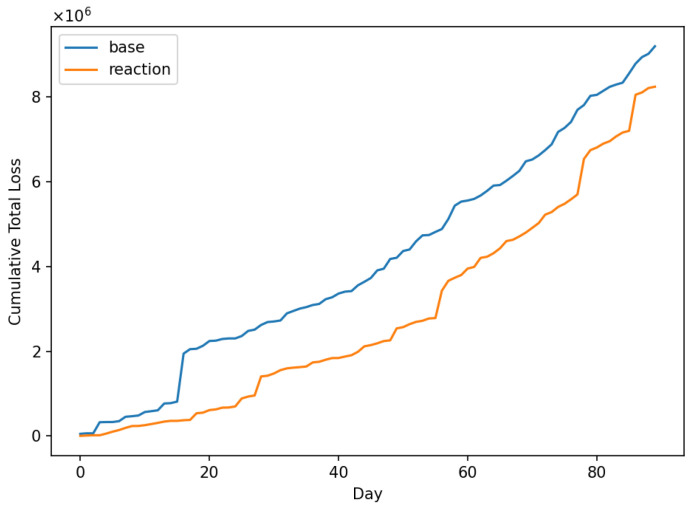
Cumulative loss: comparison of the shock and reaction to the shock.

We applied the shock of credential stuffing wave and then we utilised the reactive policy levers (Table 2). The TPP/min. rate was reduced from 600 to 300, and the user/min. rate was also reduced from 30 to 10. The anomaly threshold was raised from 0.92 to 0.94, and on-boarding threshold from 0.75 to 0.90. The comparison of the shock effect and how situation changes due to reactive policy is seen in Figure 7. This rough observation is confirmed by the chart of cumulative loss (Figure 8).

Our reaction posture, which comprises tightened rate limits, elevated anomaly threshold, and on-boarding threshold, cuts the tail of daily losses by roughly 25–30% compared with the unmitigated surge—peak drops from 1.15 M to 0.85–0.9 M on the Total Loss chart. The overall spike severity is lower, especially late in the horizon (see final third of the curve). There are still multiple spikes; the policy dampens amplitude more than frequency. That is typical for rate limits and higher anomaly cut without additional bot controls/kill-switch.

The selected KPIs confirm the initial observations. E.g., mean daily loss decreased by 25% (from 123.62 to 92.75), while ${\mathrm{VaR}}_{99.5\%}$ by 21% (from 343.69 to 270.54) and ${\mathrm{ES}}_{99.5\%}$ by 25% (from 1134.15 to 852.27). We can conclude that the reaction posture trimmed the tail risk and average losses meaningfully, but the frequency changes are modest.

## 8. Discussion and Future Research Directions

The simulation results provide clear evidence that risks in open-banking ecosystems emerge from the interaction of technological, organisational, and behavioural dynamics rather than from isolated failures. This finding aligns with prior studies highlighting that data-sharing architectures and Third-Party Provider (TPP) integrations create interdependent risk channels whose behaviour cannot be captured through static or single-actor models [3,26,27,50]. The observed divergence between incident frequency and tail-loss reductions—where enhanced controls reduce extreme losses even when overall incident counts decline only modestly—supports the working hypothesis that severity, rather than frequency, drives systemic exposure in open-banking scenarios. This pattern mirrors established findings in operational-risk and cyber-risk research, where low-frequency, high-severity events with heavy-tailed loss distributions disproportionately shape aggregate losses and capital requirements [84,85,86,87].

The models also reveal structural tensions embedded within regulatory frameworks such as PSD2. While Strong Customer Authentication and regulated API standards improve baseline security, they also introduce user-friction effects that influence adoption trajectories and TPP behaviour. The results demonstrate that compliance measures can shift incentives in ways that are not immediately visible at the policy-design stage. Moreover, the feedback loops linking ecosystem trust, user attrition, and adversarial incentives illustrate how behavioural responses can either amplify or dampen operational shocks over time.

The multi-method framework demonstrates clear advantages over single-method approaches by capturing cross-layer interactions: system-level feedback mechanisms (via System Dynamics), heterogeneous actor behaviour (via Agent-Based Modelling), and uncertainty in loss outcomes (via Monte Carlo simulation). These dimensions jointly explain why certain interventions—such as stricter TPP onboarding requirements or tighter API rate limits—produce disproportionately large reductions in loss severity. Such measures constrain adversarial leverage and reduce the conditions under which compounding failures become plausible. This provides empirical support for calls for integrated and dynamic risk-assessment tools capable of analysing risk propagation rather than point-in-time vulnerabilities. Overall, the findings suggest that open-banking resilience depends on the coordinated design of technical controls, supervisory oversight, and ecosystem governance.

Rather than prescribing specific management actions, the framework supports managerial understanding by translating model outputs into established banking risk categories. The framework primarily addresses operational risk by quantifying losses arising from failed processes, cyber incidents, fraud, and third-party dependencies associated with API-enabled open-banking services. Operational risk is measured using indicators such as incident frequency, fraud rates, unauthorised transaction loss rates, and tail-loss metrics derived from simulated loss distributions. Liquidity risk is captured indirectly through the short-term cash-flow implications of operational and fraud losses, which may generate funding stress even in otherwise well-capitalised institutions. Credit-risk effects arise through exposure channels related to customer behaviour and ecosystem concentration, proxied by open-banking adoption rates and market concentration measures. Solvency, or bankruptcy risk, is assessed through stability indicators linking accumulated operational losses to capital buffers and earnings volatility.

In practical terms, the framework is intended to support managerial decision-making rather than algorithmic optimisation. The risk measures inform the prioritisation and calibration of API rate limits, anomaly-detection thresholds, incident-response capacity, and third-party onboarding criteria. The observed reductions in tail losses indicate that targeted controls can materially lower extreme-loss exposure even when average incident frequencies remain largely unchanged—an insight that is particularly relevant for capital planning, operational-risk scenario analysis, and stress testing. By expressing open-banking risks in familiar risk categories and metrics already embedded in bank governance and supervisory processes, the framework complements existing operational-risk management, ICAAP, and supervisory stress-testing practices, while leaving final judgments and risk-appetite decisions firmly with bank management.

The general hybrid model framework proposed in this paper lays the groundwork for diverse future research avenues. Minimal modifications to the model facilitate the investigation of scenarios extending beyond the case described in Section 7. Notable examples include an adoption surge after a major fintech launch, a compromised TPP and data exfiltration, PISP mule-ring payment fraud, or a regulatory step-up (e.g., penalty multipliers and reporting SLAs). Furthermore, the model can be expanded to incorporate specific components of the “composite control index,” such as Strong Customer Authentication (SCA), Rate Throttling (RT), Anomaly Detection (AD), Behavioural Detection (BD), and Incident Response (IR). Consequently, the model could support decision-making regarding the allocation of annual budget *B* across control components to maximise risk reduction per unit of cost, subject to business impact constraints.

Beyond these structural extensions, future research should focus on calibrating the framework with field data and incorporating adaptive adversaries or machine-learning-based detection mechanisms. It would also be valuable to analyse cross-border regulatory differences, particularly in jurisdictions where open-finance frameworks extend beyond the scope of PSD2. Finally, expanding the model to capture interactions with BigTech platforms or non-bank financial intermediaries would further enhance its relevance as open-finance ecosystems continue to grow in scale and complexity.

## 9. Conclusions

This study develops an integrated modelling framework to address the core challenge of managing risk in open-banking ecosystems characterised by feedback-driven dynamics, heterogeneous actors, and uncertainty. By combining System Dynamics (SD), Agent-Based Modelling (ABM), and Monte Carlo simulation, the framework captures mechanisms of risk propagation that cannot be observed using a single methodological approach. The results show that targeted interventions, such as enhanced anomaly detection, tightened rate-limits, and more selective Third-Party Provider (TPP) onboarding, substantially reduce tail-loss exposure despite only modest effects on incident frequency, underscoring the importance of focusing on severity-reducing controls in open-banking environments.

The framework contributes a structured basis for scenario analysis, enabling the evaluation of shocks to attack intensity, ecosystem trust, API performance, control maturity, or TPP entry patterns. It also offers practical value for financial institutions and regulators seeking to prioritise risk-mitigation strategies in line with PSD2 compliance and broader operational-resilience objectives. Overall, the findings emphasise that effective risk management in open banking requires integrated, dynamic, and ecosystem-level approaches rather than siloed or static assessments.

The proposed platform is characterised by high flexibility and openness, making it a suitable starting point for a wide range of extensions. Modifying and expanding the model to enhance its precision and account for the broadest possible scope of phenomena and threats can serve as a basis for practical decision support applications, both at the system level and for individual entities.

## Figures and Tables

**Table 1:** Default values of the model parameters.

Category	Symbol	Default
Adoption and Volume	α	0.006
	φ	0.001
	$q_{0}$	3.0
	κ	0.1
	$U_{\max}$	$10^{6}$
Incidents	*A*	$10^{-7}$
	η	1.3
Controls	τ	0.02
	$\delta_{C}$	0.002
	G(z)	$0.000005\sqrt{z}$
Detection	μ(C)	0.03+0.2C
Trust	χ	0.02
	ψ	0.01
Budget	$\Pi_{\mathrm{in}}$	1000 (per day)
	Invest(B)	min(0.01B,5000)
TPP	$\rho_{\mathrm{onb}}$	0.02
	$\rho_{\mathrm{off}}$	0.005
	$I^{*}$	20

**Table 2:** Policy levers.

Scenario	SCA Level	TPP/min Limit	User/min Limit	Anomaly Threshold	On-Boarding Threshold
Default	Strong	600	30	0.92	0.75
Shock	Strong	600	30	0.92	0.75
Reaction	Strong	300	10	0.94	0.90

**Table 3:** Shock parameters.

Scenario	Attacker Multiplier	Credential Leak Active
Default	1	No
Shock	3.5	Yes
Reaction	3.5	Yes

## Data Availability

Data are contained within the article.

## Appendix A

**Table A1:** System dynamics variables and parameters.

Symbol	Domain	Meaning
*U*	≥0	Number of active users.
$U_{\max}$	>0	Potential market size.
*R*	[0,1]	Reliability index.
$R_{0}$	[0,1]	Baseline/target reliability.
*C*	[0,1]	Control maturity level.
*I*	≥0	Incident backlog.
*B*	≥0	Operational/control budget.
*P*	≥0	Number of third-party providers (TPPs).
$P_{\max}$	>0	Maximum possible TPPs.
*L*	≥0	Loss reserves.
α	>0	User adoption rate.
φ	>0	User attrition coefficient.
$f_{0}$	>0	Baseline friction in user journey.
$f_{1}$	>0	Friction increase per unit *C*.
ψ	>0	Reliability improvement rate.
χ	>0	Reliability erosion per incident backlog.
$\kappa_{R}$	>0	Decay of reliability toward $R_{0}$.
$s_{0}$	R	Baseline service level.
$s_{1}$	R	Service level gain per unit *C*.
δ	>0	Control maturity improvement rate.
ξ	>0	Control obsolescence rate.
ι	>0	Investment proportionality factor.
$\mu_{0}$	>0	Baseline incident resolution rate.
$\mu_{1}$	>0	Resolution improvement per unit *C*.
*A*	>0	Scaling factor for incident intensity.
η	>0	Sensitivity to residual risk (1−C).
*S*	>0	Scenario/stress multiplier.
*M*	>0	Exposure/monitoring multiplier.
$q_{0}$	>0	Baseline transactions per user.
$\kappa_{T}$	>0	TPP saturation parameter.
$\pi_{0}$	R	Baseline business inflow.
$\pi_{U}$	R	User-driven inflow coefficient.
$\pi_{T}$	R	TPP-driven inflow coefficient.
$\pi_{R}$	>0	Reserve-allocation rate.
$s_{C}$	>0	Cost of maintaining controls.
$s_{I}$	>0	Cost of handling incidents.
θ	>0	Adjustment speed toward target VaR.
$v_{0}$	R	Baseline VaR component.
$v_{\lambda }$	R	VaR sensitivity to incident intensity.
$v_{I}$	R	VaR sensitivity to incident backlog.
$\gamma_{\lambda }$	>0	Loss per incident intensity.
$\gamma_{I}$	>0	Loss per incident backlog.
${\mathrm{VaR}}^{*}$	>0	Target Value-at-Risk.

**Table A2:** ABM and Monte Carlo variables and parameters.

Symbol	Domain	Meaning
$M=[m_{b,j}]$	{0,1}	Bank–TPP connection matrix.
$W=[w_{b,j}]$	≥0	Traffic-weight matrix.
$p_{\mathrm{connect}}$	[0,1]	Connection probability.
Q(k)	≥0	Total transaction volume on day *k*.
$c_{j}\left(k\right)$	≥0	Transaction load of TPP *j*.
$w_{j}\left(k\right)$	[0,1]	Traffic share of TPP *j*.
$\lambda_{\mathrm{att}}$	>0	Attack intensity coefficient.
$p_{0}$	(0,1)	Baseline attack success probability.
γ	>0	Traffic sensitivity of attack success.
$K_{\mathrm{thr}}$	>0	Fraud threshold for throttling.
ρ	(0,1]	Traffic fraction removed on throttling.
N(t)	N	Number of loss events.
Λ(t)	>0	Poisson rate for event frequency.
λ(t)	>0	Event intensity (Poisson mode).
$X_{i}\left(t\right)$	>0	Loss severity of event *i*.
ℓ(t)	≥0	Total loss on day *t*.
*M*	N	Number of Monte Carlo iterations.
$\mu_{t}$	R	Lognormal location parameter.
$\sigma_{t}$	>0	Lognormal scale parameter.
$\mu_{0}^{X}$	R	Baseline severity intercept.
$\mu_{1}^{X}$	R	Sensitivity to lnQ(t).
$\mu_{2}^{X}$	R	Severity reduction per unit *C*.
*q*	(0,1)	Confidence level for VaR/ES.
